# Loss of ATRX, associated with DNA methylation pattern of chromosome end, impacted biological behaviors of astrocytic tumors

**DOI:** 10.18632/oncotarget.3906

**Published:** 2015-05-04

**Authors:** Jinquan Cai, Jing Chen, Wei Zhang, Pei Yang, Chuanbao Zhang, Mingyang Li, Kun Yao, Hongjun Wang, Qingbin Li, Chuanlu Jiang, Tao Jiang

**Affiliations:** ^1^ Department of Neurosurgery, The Second Affiliated Hospital of Harbin Medical University, Harbin, China; ^2^ Beijing Neurosurgical Institute, Capital Medical University, Beijing, China; ^3^ Beijing Institute for Brain Disorders Brain Tumor Center, Beijing, China; ^4^ Department of Neurosurgery, Beijing Tiantan Hospital, Capital Medical University, Beijing, China; ^5^ China National Clinical Research Center for Neurological Diseases, Beijing, China; ^6^ Chinese Glioma Cooperative Group (CGCG), Beijing, China; ^7^ Department of Pathology, Beijing Sanbo Brain Hospital, Capital Medical University, Beijing, China

**Keywords:** ATRX, DNA methylation, chromosome end, MGMT, biological behaviors

## Abstract

Loss of ATRX leads to epigenetic alterations, including abnormal levels of DNA methylation at repetitive elements such as telomeres in murine cells. We conducted an extensive DNA methylation and mRNA expression profile study on a cohort of 82 patients with astrocytic tumors to study whether *ATRX* expression was associated with DNA methylation level in astrocytic tumors and in which cellular functions it participated. We observed that astrocytic tumors with lower *ATRX* expression harbored higher DNA methylation level at chromatin end and astrocytic tumors with *ATRX*-low had distinct gene expression profile and DNA methylation profile compared with *ATRX*-high tumors. Then, we uncovered that several *ATRX* associated biological functions in the DNA methylation and mRNA expression profile (GEP), including apoptotic process, DNA-dependent positive regulation of transcription, chromatin modification, and observed that *ATRX* expression was companied by MGMT methylation and expression. We also found that loss of ATRX caused by siRNA induced apoptotic cells increasing, reduced tumor cell proliferation and repressed the cell migration in glioma cells. Our results showed *ATRX*-related regulatory functions of the combined profiles from DNA methylation and mRNA expression in astrocytic tumors, and delineated that loss of ATRX impacted biological behaviors of astrocytic tumor cells, providing important resources for future dissection of *ATRX* role in glioma.

## INTRODUCTION

Astrocytic tumors are the most common primary malignant brain tumors in adults [1]. They are heterogeneous in cellular composition consisting of tumor stem cells, mesenchymal cells, and host stromal cells. Primary glioblastoma can arise de novo, whereas secondary glioblastoma is thought to arise from lower-grade gliomas [2]. Lots of studies have resulted in the identification of various chromosomes and genes that are frequently altered in astrocytic tumours [3].

Mutations in X-linked alpha thalassaemia mental retardation (*ATRX*), encoding a SWI/SNF-like protein, were first identified in patients bearing the X-linked alpha thalassemia/mental retardation syndrome [4, 5]. *ATRX* plays a variety of key role at tandem repeat sequences within the genome, including prevention of replication fork stalling, the deposition of a histone variant, and the suppression of a homologous recombination-based pathway of telomere maintenance [6–8]. Recent reports described that *ATRX* mutation or loss occurred at high percentages in multiple tumor types, including low grade astrocytoma and secondary glioblastoma, suggestive of a potential “driver” role in cancer [9–11]. And several teams demonstrated that *ATRX* alteration, combined with other classical biomarkers, refined the molecular classification of adult gliomas, providing a prognostic tool for clinicians [12–14]. In addition, loss of ATRX leads to epigenetic alterations, including abnormal levels of DNA methylation at repetitive elements such as telomeres in murine cells [15]. Epigenetic alterations are now accepted as having a role in tumorigenesis [16]. DNA methylation alterations have been widely reported in human glioblastoma multiform (GBM) and other glioma subtypes [17, 18]. In 2013, from The Cancer Genome Atlas (TCGA)'s report, GBMs harboring *ATRX* mutation were enriched in G-CIMP + subtype [3]. In current work, we aimed to study whether *ATRX* expression was associated with DNA methylation level in astrocytic tumors and in which cellular functions it participated. We conducted an extensive DNA methylation and mRNA expression profile study on a cohort of 82 patients with astrocytic tumors in China. We observed that astrocytic tumors with lower *ATRX* expression harbored higher DNA methylation level at chromatin end and astrocytic tumors with *ATRX*-low had distinct GEP and DNA methylation profile compared with *ATRX*-high tumors. Then, we uncovered that several *ATRX* associated biological functions in the DNA methylation and mRNA expression profile, for example, apoptotic process, metabolic process, DNA-dependent positive regulation of transcription chromatin and modification. Interestingly, we observed that astrocytic tumors with lower *ATRX* expression companied with *MGMT* (O^6^-methylguanine–DNA methyltransferase) hypermethylation and downregulation. Consistent with our report [14], *ATRX* expression characteristically decreased in grade II astrocytomas and secondary glioblastoma and low *ATRX* expression was correlated with favorable survival of patients in astrocytic tumors. And we also found that loss of ATRX caused by siRNA induced apoptotic cells increasing, reduced tumor cell proliferation and repressed the cell migration in glioma cells. Our results provided novel insights into *ATRX*-related regulatory functions at the DNA methylation and mRNA expression level in astrocytic tumors, and here could serve as important resources for future dissection of *ATRX* role in glioma.

## RESULTS

### *ATRX* mRNA expression characteristically decreased in grade II astrocytomas and secondary glioblastoma

**Figure 1:**
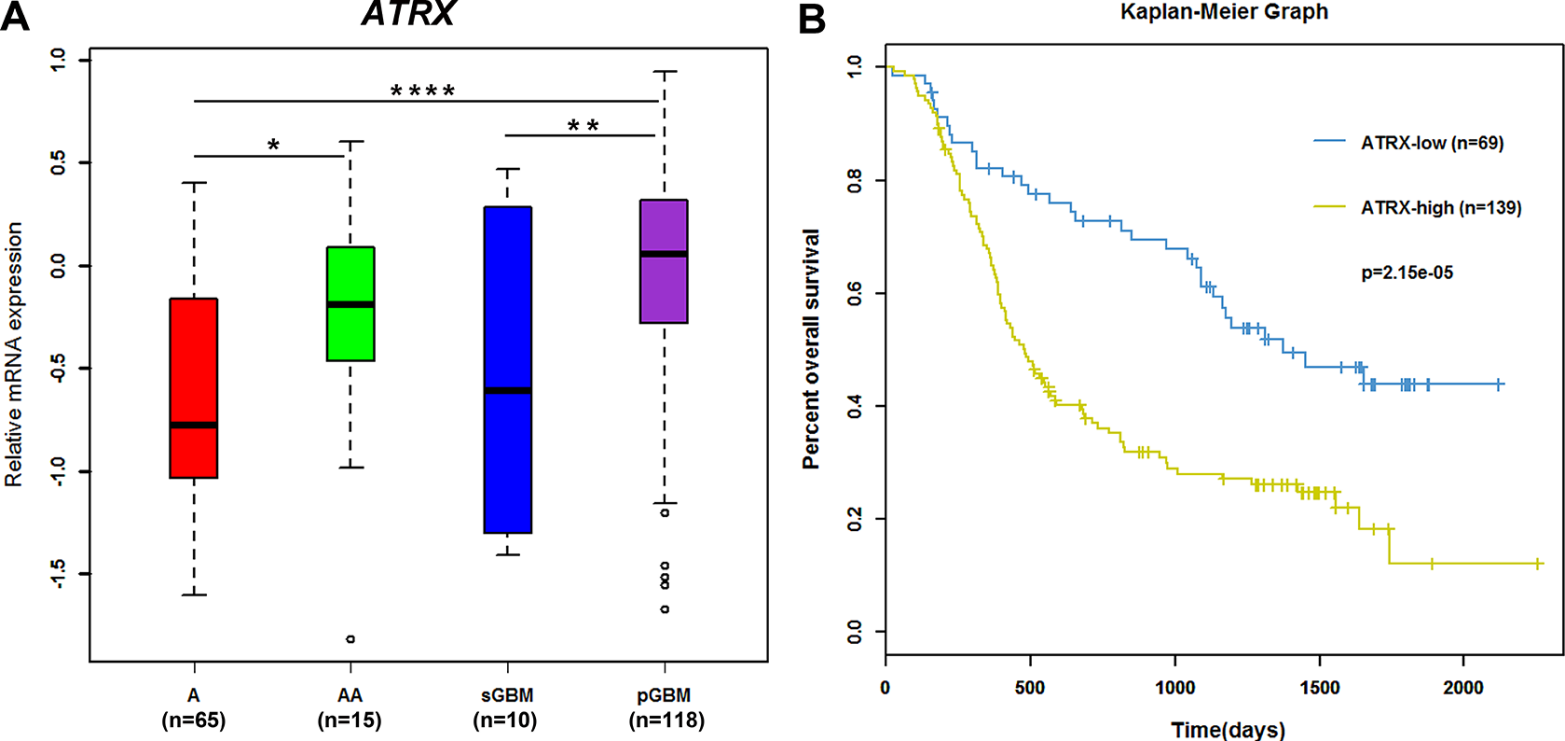
*ATRX* mRNA expression correlated with tumor grade and survival in astrocytic tumors *ATRX* mRNA expression was significantly different in grade II-IV astrocytic tumors (**A.** *p* < 0.0001) and reduced in grade II astrocytomas than in pGBMs and AA (**A.** *p* < 0.0001, *p* < 0.05, respectively). Secondary GBMs harbored lower *ATRX* expression compared with primary GBMs (Figure 1A; *p* < 0.01). Patients in the *ATRX*-low group displayed significantly longer overall survival (OS) than patients in the *ATRX*-high group (**B.** log-rank test, *p* = 2.15e-05).

In our mRNA array expression profile, there were 65 diffuse astrocytomas (As, Grade II), 15 anaplastic astrocytomas (AAs, Grade III), 10 secondary glioblastomas (sGBMs, Grade IV) and 118 primary glioblastomas (pGBMs, Grade IV). *IDH1/2* mutations occurred in 77.4% (48/62) As, 40% (6/15) AAs, 60% (6/10) sGBMs and 12.7% (15/118) pGBMs. Consistent with our previous report, *ATRX* mRNA expression was significantly different in grade II-IV astrocytic tumors (Figure 1A; *p* < 0.0001) and reduced in grade II astrocytomas compared with in pGBMs and AAs (Figure 1A; *p* < 0.0001, *p* < 0.05, respectively). We also observed that secondary GBMs harbored lower *ATRX* expression than primary GBMs (Figure 1A; *p* < 0.01). These results suggested that *ATRX* expression was associated with malignancy in astrocytic tumors.

### *ATRX* mRNA expression was correlated with survival in patients with astrocytic tumors

We defined the *ATRX*-high group and the *ATRX*-low group according to the cutoff point (−0.5368) (Supplementary Figure 1). There were 69 samples, harboring lower *ATRX* expression than the cutoff point, in the *ATRX*-low group, which included 41 As, 4 AAs, 5 sGBMs and 19 pGBMs. The *ATRX*-high group contained 24 As, 11 AAs, 5 sGBMs and 99 pGBMs (Supplementary Table 1). Decreased *ATRX* expression was associated with favorable survival of patients with astrocytic tumors (Figure 1B; *p* = 2.15e–05). To control the influence of age at diagnosis, gender, preoperative Karnofsky performance status (KPS) score, extent of surgical resection, chemotherapy, and radiotherapy on the stratification of astrocytic gliomas, we performed the Cox regression model as showed in Supplementary Table 2. The prognostic value of *ATRX* expression was still significant, independent of age at diagnosis, preoperative KPS score, extent of surgical resection, and radiotherapy.

### *ATRX*-low astrocytic tumors had distinct GEP and DNA methylation profile compared with *ATRX*-high tumors

**Figure 2:**
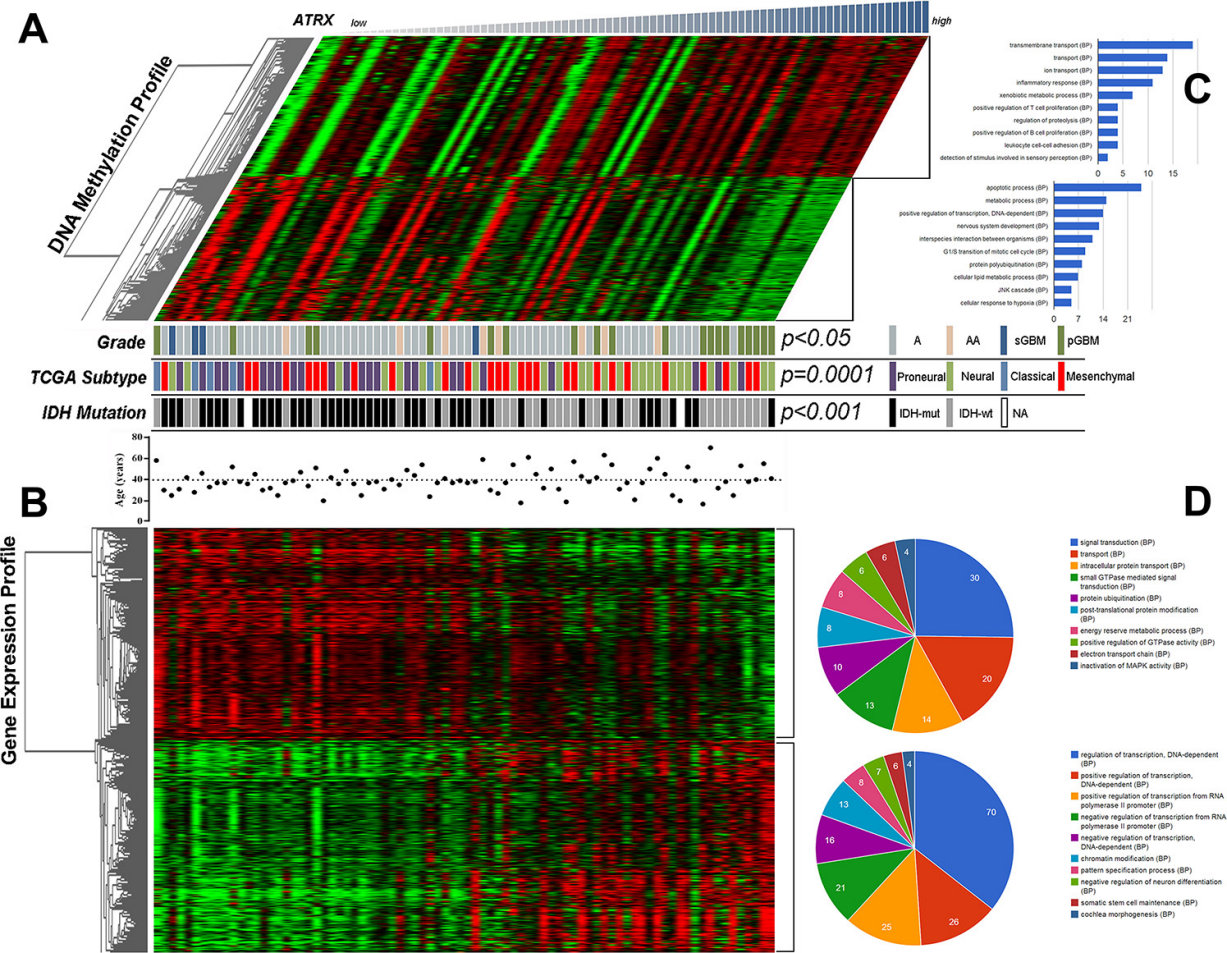
Clustering analysis of both DNA methylation and mRNA expression profiles in astrocytic tumors Patterns of mRNA expression and DNA methylation were associated with *ATRX* in astrocytic tumors using one-dimensional hierarchical clustering analysis **A.** and **B.** Functional enrichment analysis of associated genes, indicating the functional roles of gene sets in different subgroups. Enrichment results for biological processes were obtained from the GO database. The orders of biological processes listed in the circle or histogram are based on the number of targets annotated in the biological process **C.** and **D.**

Matched genome-wide mRNA expression and DNA methylation profile was successfully obtained from these 82 astrocytic tumor samples (Table 1). To identify which genes methylation and expression level likely associated with *ATRX* expression in astrocytic tumors, pearson correlation analysis was performed, respectively. We screened top 500 positively correlated expression probes (*r* > 0.525682177, *p* < 3.95e–07) with *ATRX* and top 500 negatively correlated expression probes (*r* < −0.505967751, *p* < 1.24e–06). On the other hand, top 300 positively correlated methylation probes (*r* >0.340059, *p* < 0.001773) with *ATRX* and top 300 negatively correlated with methylation probes (*r* < −0.37802, *p* < 0.000463) were screen. Expression and methylation patterns of genes correlated with *ATRX* were showed using one-dimensional hierarchical clustering analysis in Figure 2A/2B. The four most representative biological processes for genes commonly altered by hypermethylation were apoptotic process (for example, *TNFSF10* and *TNFRSF19*), metabolic process (for example, *PDHA1* and *UCK1*), DNA-dependent positive regulation of transcription (for example, *BRCA1* and *RARA*) and nervous system development (for example, *METRN* and *DBN1*) (Figure 2C). On the other hand, these genes regulated by hypomethylation in *ATRX*-low tumors were implicated with transport, including transmembrane transport and ion transport, and immune response, including inflammatory response, positive regulation of T cell proliferation, positive regulation of B cell proliferation and leukocyte cell-cell adhesion (Figure 2C). Among the genes involved in immune response, *IL23R, ITGAL, CCL22* and *IL22* were hypomethylated.

**Table 1:** Clinicopathological characteristics of 82 patients with astrocytic tumors

Characteristic	No. of Patients
***Age at diagnosis***	
**< 45**	58
**≥ 45**	24
***Gender***	
**Male**	50
**Female**	32
***Preoperative KPS score***	
**≥ 80**	49
**< 80**	20
***Histology & Grade***	
**Astrocytoma (A, II)**	50
**Anaplastic astrocytoma (AA, III)**	8
**Primary glioblastoma (pGBM, IV)**	20
**Secondary glioblastoma (sGBM, IV)**	4
***TCGA Subtypes***	
**Proneural**	24
**Neural**	26
**Classical**	5
**Mesenchymal**	27
***Resection***	
**Total**	31
**Subtotal**	47
***Radiotherapy***	
**Yes**	61
**No**	15
***TMZ Chemotherapy***	
**Yes**	27
**No**	40
***IDH1/2 mutation***	
**Mutation**	46
**Wildtype**	34

Abbreviations: KPS, Karnofsky performance status.

A similar analysis approach was taken to investigate these correlated expression gene probes. One-dimensional hierarchical clustering resulted in a good separation between *ATRX*-low and *ATRX*-high (Figure 2B). These genes overexpressed in *ATRX*-low tumors also participated in transport and protein modification process, especially intracellular protein transport, protein ubiquitination and post translational protein modification, and signal transduction including small GTPase mediated signal transduction and positive regulation of GTPase activity. In addition, regulation of transcription and chromatin modification were the most frequently deregulated biological process (Figure 2D). In this respect, a total of 13 genes involved in the chromatin modification, including *BANP, PHF2, FOXA2, HMG20B, CABIN1, DOT1L, CHD8, KDM5A, CCDC101, EP400, EPC1, KDM5*B and *USP3*.

Moreover, we observed that proneural or *IDH*-mut tumors mainly enriched in the *ATRX*-low group (*p* = 0.0001 and *p* < 0.001, respectively).

### Decreased *ATRX* expression was associated with DNA methylation level of chromatin end

**Figure 3:**
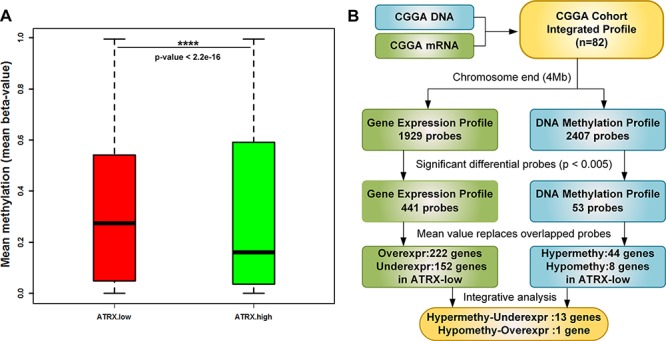
Strategy to identify significant genes of both DNA methylation and gene expression profiles According to Sturm et al.'s work [18], we screened the probes within 4Mb bases to chromosome end. Mean methylation value within 4 Mb to the chromosome end normalized to the mean overall methylation value. Significant differences (*p* < 2.2e-16) between these two subgroups are indicated **A.** Tumors in *ATRX*-low cluster specifically showed widespread hypermethylation at chromosome end compared with *ATRX*-high subgroup.1929 probes from gene expression profile and 2407 probes from DNA methylation profile were analyzed, respectively at the chromosome end. We identified that 441 differential probes from gene expression profile and 53 differential probes from DNA methylation profile (*p* < 0.005). Comparison of the 52 putative target genes of differential methylation and the 374 genes of differential expression allowed the detection of gene loci that experienced both concurrent changes in *ATRX*-low tumors. In total, 18 genes were both differentially methylated and differentially expressed: 17 of these were hypermethylated, and 1 were hypomethylated. Thirteen of the genes that were hypermethylated in *ATRX*-low also featured downregulated gene expression **B.**

According to Sturm et al's work [18], we screened the probes within 4Mb bases to chromosome end, and then we observed that tumors in *ATRX*-low cluster specifically showed widespread hypermethylation at chromosome end, when compared with *ATRX*-high subgroup, potentially linking subtelomeric methylation pattern to alternative lengthening of telomeres (ALT) (Figure 3A). 1929 probes from gene expression profile and 2407 probes from DNA methylation profile were analyzed, respectively at the chromosome end (Figure 3B). Significantly differential probes (*p* < 0.005) were screened via t test. 441 probes from gene expression profile and 53 probes from DNA methylation profile were identified. Multiple probes were corresponding to a single a gene, the expression/methylation values of these redundant probes were averaged. Integrative analysis involving methylation and expression profiling was used to characterize genomic changes between *ATRX*-low tumors and *ATRX*-high tumors. Comparison of the 52 putative target genes of differential methylation and the 374 genes of differential expression allowed the detection of gene loci that experienced both concurrent changes in ATRX-low tumors. In total, 18 genes were both differentially methylated and differentially expressed: 17 of these (94%) were hypermethylated, and 1 (6%) were hypomethylated. Among them, a high proportion of hypermethylated genes was downregulated. Thirteen of the genes that were hypermethylated in ATRX-low also featured downregulated gene expression (76%). Using functional enrichment, we observed that cell death (CLN8, PANK2, BNIP3) and DNA repair (TYMS, MGMT) were the most significant-represented terms (Table 2).

**Table 2:** Integrative analysis of hypermethylated and underexpressed genes in ATRX-low patients

Gene symbol	*Expression*				*Methylation*			
	ATRX-low	ATRX-high	fold change	*p* value	ATRX-low	ATRX-high	fold change	*p* value
**GAS8**	0.8874	1.3309	0.6668	0.0001	0.3448	0.2098	1.6432	0.0001
**MGMT**	1.8678	2.5767	0.7249	0.0000	0.1869	0.1122	1.6662	0.0007
**CLN8**	1.8429	2.3516	0.7837	0.0016	0.2118	0.1093	1.9379	0.0010
**LMBR1**	2.4871	2.8192	0.8822	0.0046	0.2236	0.1321	1.6932	0.0014
**BNIP3**	1.4292	1.7909	0.7980	0.0020	0.2122	0.1149	1.8470	0.0016
**LY6E**	0.9572	1.4992	0.6385	0.0003	0.2377	0.1251	1.8994	0.0020
**ZFP28**	1.4123	2.1194	0.6663	0.0018	0.2386	0.1653	1.4439	0.0022
**GLT1D1**	2.7025	3.9834	0.6784	0.0001	0.3092	0.2020	1.5309	0.0030
**TYMS**	2.6947	3.6345	0.7414	0.0029	0.2429	0.1472	1.6504	0.0035
**ANKRD11**	2.4346	2.7741	0.8776	0.0011	0.1684	0.0889	1.8943	0.0036
**PANK2**	1.4702	1.7699	0.8307	0.0021	0.1469	0.0663	2.2139	0.0037
**TUBB2A**	1.9709	3.0462	0.6470	0.0000	0.3389	0.2299	1.4743	0.0045
**PRKAR1B**	0.9218	1.5021	0.6137	0.0002	0.2416	0.1342	1.8002	0.0048

**Figure 4:**
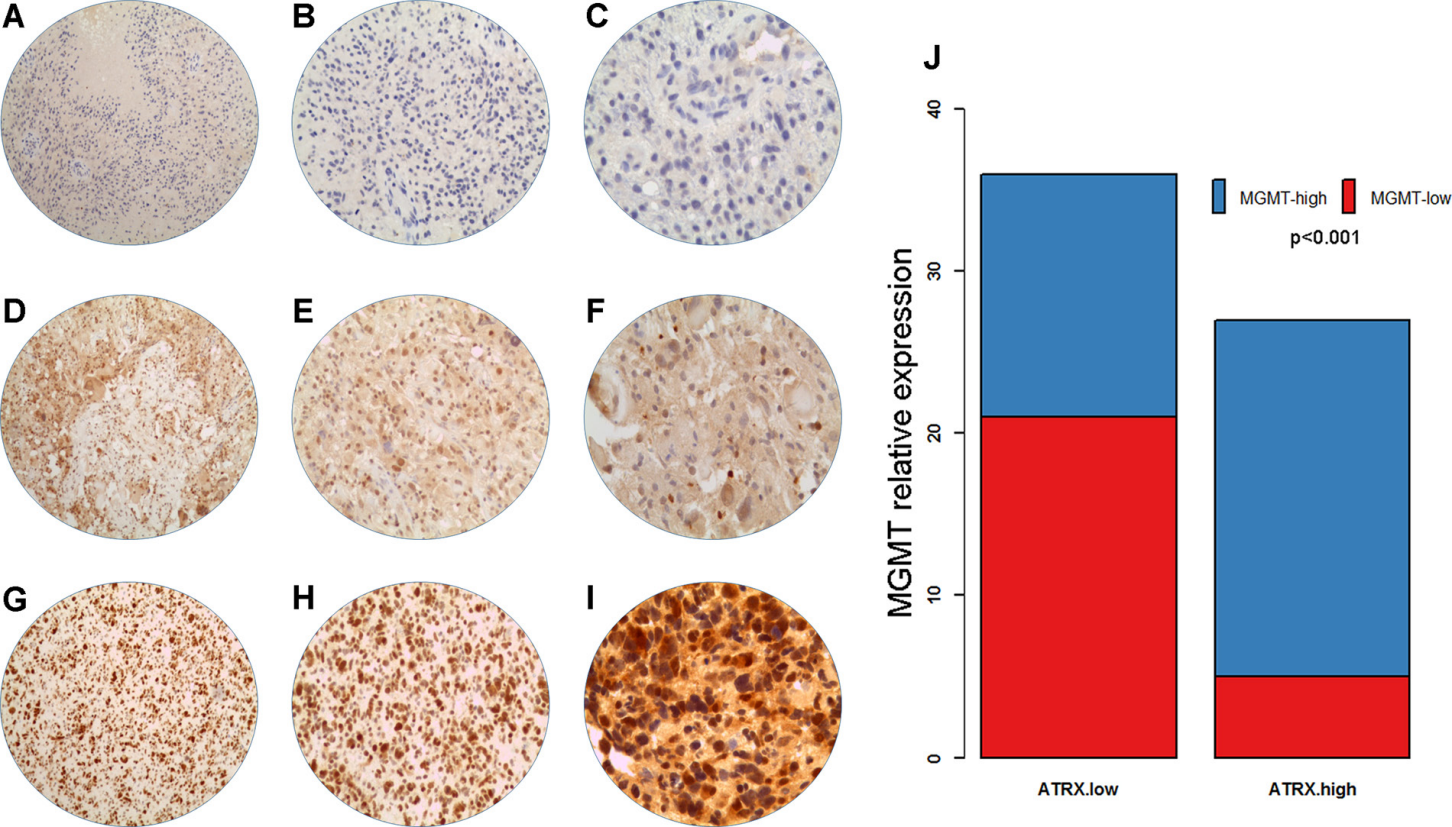
MGMT expression by immunohistochemical staining Negative (**A.** 100 ×; **B.** 200 ×; **C.** 400 ×); Weakly positive (**D.** 100 ×; **E.** 200 ×; **F.** 400 ×); Strongly positive (**G.** 100 ×; **H.** 200 ×; **I.** 400 ×). *ATRX*-low astrocytic tumors harbored lower MGMT expression (**J.** *p* < 0.001).

To validate the association between *ATRX* and MGMT expression, we detected the MGMT protein expression via immunohistochemistry. We observed that ATRX-low astrocytic tumors harbored lower MGMT expression (Figure 4).

### The knockdown of ATRX affected migration, apoptosis and proliferation of LN229 cells

**Figure 5:**
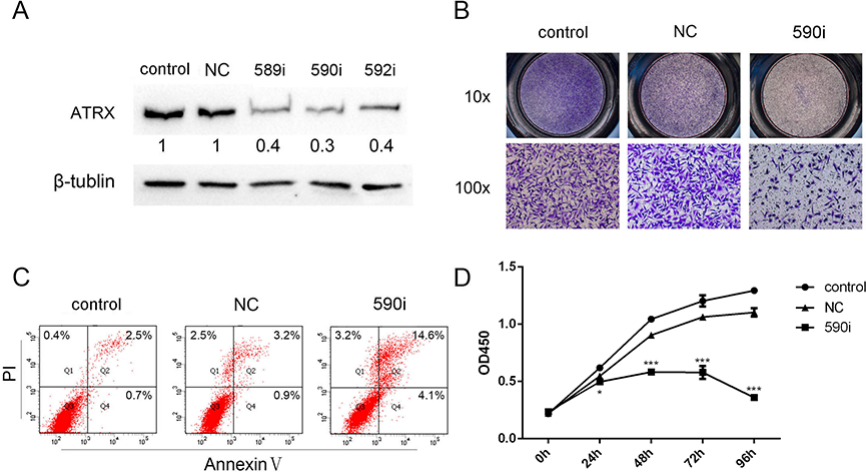
The knockdown of ATRX affected migration, apoptosis and proliferation of LN229 cells The expression levels of ATRX were detected by western blotting in LN229 cells that were transiently transfected with negative control (NC), 589i, 590i and 592i. β-tublin was used as a control for equal protein loading. The bands were scanned using Image Lab 5.1 software. The densitometric values normalized to β-tublin expression are indicated below the corresponding lanes, and are shown as the fold change relative to that in the LN229 cells transfected with negative control **A.** Contrasted with control and NC, notably less LN229 cells transfected with 590i penetrated the transwell at 24 h after cells seeded. (up) 10 × ; (down) 100 × **B.** Flow cytometry analysis of Annexin V and PI levels in the LN229 cells untreated or transfected with NC or 590i. Results are a representative experiment of the three conducted. The percentages of Annexin V and PI-positive cells are shown **C.** Transfection of LN229 cells with 590i inhibited cell proliferation **D.** *p < 0.05, **p < 0.01 and ***p < 0.001.

In the present study, to examine effect of *ATRX* on glioma cells, the intrinsic expression of ATRX was repressed by siRNA 589i, 590i and 592i. Repression efficiency was confirmed by western blot analysis (Figure 5A) and real-time PCR (Supplementary Figure 2A), which indicated that ATRX expression inhibition was the most significant in 590i transfected cells. The effect on migration of ATRX was verified by transwell chamber assay. Cell apoptosis was monitored by evaluating the percentage of Annexin V-positive and PI-positive cells by flow cytometry. Cell counting kit-8 (CCK-8) assay was performed to identify cell viability. The results of the present study showed that *ATRX* siRNA 590i markedly reduced LN229 cells migration, but in contrast, the negative control and untransduced had no inhibitory effect on it (Figure 5B and Supplementary Figure 2B). Following 590i transfection, flow cytometry analysis revealed a significantly higher percentage of Annexin V- and PI-positive cells among the LN229 cells (Figure 5C and Supplementary Figure 2C) compared with the cells transfected with negative control. CCK-8 assay showed that the knockdown of ATRX inhibited cells proliferation. The inhibitory effect of 590i was significant at 24 h, and it was maximal at 48 h, 72 h and 96 h (Figure 5D). These results indicated that reduced expression of ATRX inhibited the migration, promoted apoptosis and reduced proliferation of LN229 cells. This suggested important role of *ATRX* for glioma cells.

## DISCUSSION

Aberrant gene function and altered patterns of gene expression are key features of glioma [18]. Increasing evidences showed that acquired epigenetic abnormalities participated with genetic alterations to cause this dysregulation [16]. Localized hypermethylation of gene-associated CpG islands and a more extensive genome-wide reduction in 5-methylcytosine are epigenetic events that typify many tumors [19]. Methylation is the known covalent modification of genomic DNA in humans and occurs at cytosines followed by guanines (CpG) [20]. Aberrant methylation of some CpG islands in cancer has been associated with silencing of tumor suppressor genes [21]. According to the TCGA report, patients with G-CIMP tumors, a distinct subset of samples displays concerted hypermethylation at a large number of loci, experience significantly improved outcome [17]. An important link between DNA methylation and these covalent histone modifications was illustrated by the pioneering experiments of Nan et al. [22]. Mutations in *ATRX,* encoding a SWI/SNF-like protein, lead to changes in the pattern of methylation of several highly repeated sequences including the rDNA arrays, a Y-specific satellite and subtelomeric repeats [15]. In the present study, we integratively analyzed genome-wide DNA methylation and mRNA expression profiling of 82 astrocytic tumor samples. We screened top 500 positively/negatively correlated expression probes with *ATRX* and top 300 positively/negatively correlated CpG locis with *ATRX*. We observed that *ATRX*-low astrocytic tumors had distinct GEP and DNA methylation profile compared with *ATRX*-high tumors. To our knowledge, this is the first time the same cohort of patients with both profiles has been used to analyze *ATRX* associated functions in astrocytic tumors. *ATRX* played a key role in the incorporation of the histone variant H3.3 into pericentromeric DNA, ribosomal and telomeric [8]. In addition, *ATRX* functioned in maintaining telomere integrity and facilitating normal telomere replication during DNA synthesis [6, 23]. Previous study showed that loss of SWI/SNF-mediated transcriptional activation was implicated in direct interactions with promoter sequences of affected genes and increased DNA methylation in cancer cells, providing insight into the mechanisms underlying aberrant gene induction and repression during tumor progression [24]. Our functional analysis results also confirmed that *ATRX* was associated with apoptotic process, DNA-dependent positive regulation of transcription, protein transport and chromatin modification in astrocytic tumors through Gene Ontology analysis, suggesting a functional role of *ATRX* alteration in astrocytic tumors development. Then, we compared the mean methylation level of chromosome end between *ATRX*-low tumors and *ATRX*-high tumors and investigated the combined profiles of DNA methylation and mRNA expression at chromosome end. The integrative study identified DNA methylation markers that could lead to the downregulation of some genes involved in important cellular functions in *ATRX*-low tumors: *MGMT, TYMS, CLN8, PANK2* and *BNIP3*. We also identified that, in glioma cell line, the knockdown of ATRX expression induced apoptotic cells increasing, reduced tumor cell proliferation and repressed the cell migration. Similarly, a diagram depicting how loss of ATRX could affect neuroprogenitor cell apoptosis was provided by a report [25].

In conclusion, our results showed *ATRX*-related regulatory functions of the combined profiles from DNA methylation and mRNA expression in astrocytic tumors, and delineated that loss of ATRX impacted biological behaviors of astrocytic tumor cells, providing important resources for future dissection of *ATRX* role in glioma.

## MATERIALS AND METHODS

### Datasets collection

Whole genome mRNA expression profile (GEP) of 208 astrocytic tumor samples of all grades and DNA methylation profile of 105 astrocytic glioma samples, by using Agilent Whole Human Genome Array and Illumina Infinium Human Methylation 27K Bead Chip, respectively, were obtained from Chinese Glioma Genome Atlas (CGGA) database (http://www.cgga.org.cn) [26, 27]. For molecular subtype annotation of the CGGA dataset, we applied prediction analysis of microarrays (PAM) as previously reported [28]. All these samples were histologically graded according to current WHO classification of tumours of the nervous systems [29]. Written informed consent was obtained from all donors. Clinical investigations were performed after approval by the local research ethics committee and in accordance with the ethical principles.

### Molecular analysis

#### *IDH* mutation

Genomic DNA was isolated from frozen tissues with a QIAamp DNA Mini Kit (Qiagen) as the manufacturer's protocol. DNA concentration and quality were evaluated with a Nano-Drop ND-1000 spectrophotometer (NanoDrop Technologies, Houston, TX). Pyrosequencing of *IDH1/2* mutations was supported by Gene-tech (Shanghai, China) and performed on a Pyro-Mark Q96 ID System (Qiagen, Valencia, Calif). The primers 5′-GCTTGTGAGTGGATGGGTAAAAC-3′, 5′-BiotinTTGCCAACATGAC TTACTTGATC-3′ for IDH1 and 5′-ATCCTGGGGGGGACTGTCTT-3′, 5′-Biotin-CTCTCCACCCTGGCCT ACCT-3′ for IDH2 were used for PCR amplification, and the primers 5′-TGGATGGGTAAAACCT-3′ for IDH1 and 5′-AGCCCATCACCATTG-3′ for IDH2 were used for pyrosequencing [14].

### MGMT protein expression

Immunohistochemical analysis was performed as described in our previous study [30]. Briefly, tumor tissues were formalin-fixed and embedded in paraffin. For detecting MGMT expression, 5 μm sections were incubated with monoclonal antibodies against MGMT (Santa Cruz Biotechnology, Santa Cruz, CA, USA). All samples were evaluated by two pathologists experienced in analysis of tumors of the central nervous system. Scoring on the expression level of MGMT protein was performed according to the percentage of positive MGMT cells. A score of less than 5% was classified as negative MGMT expression and a score of 5–100% was classified as positive MGMT expression. Scores of 5–25% were denoted as weakly positive MGMT expression, and scores of 25–100% was denoted as strongly positive MGMT expression.

### Gene ontology (GO) and KEGG pathway analysis

After Pearson correlation analysis, gene ontology analysis or KEGG pathway of the correlated genes were analyzed by GeneCodis (http://genecodis.cnb.csic.es/) [31–33].

### Cell lines and cell culture

The human glioma cell line LN229 was obtained from the Institute of Biochemistry and Cell Biology, Chinese Academy of Science. LN229 cell line was maintained in DMEM F12 medium (Gibco), 10% FBS (Gibco), 50 units/ml penicillin and 50 μg/ml streptomycin (Sigma) at 37°C in a 5% CO2 atmosphere.

### Oligonucleotides and cell transfection

Stealth siRNA targeting *ATRX* 589i (GCAGATTGAT ATGAGAGGAAT), 590i (CGACAGAA ACTAACCCT GTAA), 592i (CCGGTGGTGAACATAAG AAAT) and negative control (UUCUCCGAACGUGUCACGUTT) was obtained from GenePharma. For the siRNA experiments, LN229 cells were transfected with either a control non-targeting human RNA or siRNA against *ATRX* at a final concentration of 200 nM. All cell transfections were introduced by the RNAi MAX reagent (Invitrogen) according to the manufacturer's instructions. The cell transfections were performed in six-well plates. For each transfection, 7 μl of transfection reagent was used and three replication experiments were performed.

### Western blotting and antibodies

Whole-cell lysates were prepared using RIPA buffer. Equal amounts of total protein (30 μg) from cell lysates were loaded on a 6% SDS/PAGE gel, transferred to a PVDF membrane (Millipore), and detected using an ECL Western Blotting Detection System (Biorad). Primary antibodies were primary antibodies against ATRX (ab97508; Abcam; 1:1000), β-tublin (CW0098A; CWBIO; 1:5000). Secondary antibodies used were goat anti-rabbit IgG-HRP and goat anti-mouse IgG-HRP (Zhongshan Gold Bridge Biotechnology). Immunoblots were quantified using Image Lab 5.1 software.

### RNA isolation and quantitative real-time RT-PCR

Total RNA was extracted from the harvested cells using RNAprep pure Cell Kit (Tian Gen) according to the manufacturer's instructions. cDNA was synthesized by M-MLV (Moloney murine leukaemia virus) reverse transcriptase (Invitrogen) from 2 μg of total RNA. Oligo (dT) 18 was used as the primer for reverse transcription of mRNA. Quantitative real-time RT-PCR was carried out in a 7500 real-time PCR System (Applied Biosystems) using the SYBR Select Master Mix (Applied Biosystems) according to the manufacturer's instructions. For each mRNA assay, the data were normalized using the endogenous actin cDNA control. The comparative CT method was used to quantify the target genes relative to the endogenous control. For each individual analysis one of the samples was designated as the calibrator and given a relative value of 1.0. All quantities were expressed as then-fold relative to the calibrator. The real-time PCR primers were as follows: ATRX forward-5′ GCTGAGCCCATGAGTGAAAG 3′, reverse-5′ CGTGA CGATCCTGAAGACTTG 3′; β-actin forward-5′ CATGTA CGTTGCTATCCAGGC 3′, reverse-5′ CTCCTTAATGT CACGCACGAT 3′.

### Cell proliferation assay

LN229 cells plated at a final concentration of 104 cells /well in 96-well plates were transfected with NC or si-ATRX in exponential growth. The viability of cells was evaluated by cell counting kit-8 (CCK-8) assay after 0, 24, 48, 72 and 96 h of transfection. The optical density at 450 nm (OD450) of each well was measured with a microplate reader (Infinite^®^ M1000 PRO, TECAN).

### Cell migration assay

The lower Transwell chamber (Costar) contained cell appropriate medium (10% fetal calf serum as chemoattractants). Cells in appropriate medium (1% fetal calf serum) were seeded onto membranes of the upper Transwell chamber (6.5 mm diameter, 8 μm pores). After 48 h of transfection with NC or si-ATRX 105 LN229 cells were incubated for 24 h, cells were ethanol-fixed and stained (crystal violet). The non-invading cells were removed from the upper surfaces of the invasion membranes and the cells on four fields of the lower face were counted using an inverted microscope.

### Cell apoptosis assay

Cell apoptosis detection was performed by fluorescence-activated cell sorting (FACS) analysis using a flow cytometer. LN229 cells were placed in six-well plates, and after transfection with NC or si-ATRX for 72 h, cells were harvested. After centrifugation, cell were washed twice with cold phosphate-buffered saline which then incubated with 5 μL of annexin V-FITC and 10 μL of PI at room temperature for 15 min in the dark. After incubation, 400 μL of 1× binding buffer was added to each tube. The cells were immediately analyzed by FACS Calibur flow cytometry (Becton Dickinson).

### Statistical analysis

According to the cutoff value (−0.5368), patients in our dataset were stratified into the *ATRX*-high group and the *ATRX*-low group. The threshold was determined by receiver operating characteristics (ROC) analysis with an area under the curve of 0.6396 (Supplementary Figure 1).

We screened the probes within 4Mb bases to chromosome end as corresponding genomic coordinates of the probes according to Sturm et al's work [18]. Overall survival was estimated from the date of diagnosis to the date of either death or last follow-up. Patients were censored at the time they were last known to be alive. Kaplan-Meier analysis was performed to estimate the survival time of different subgroups and a log-rank test was used to test for differences of more than one survival curve. Student's *t*-test and analysis of variance (ANOVA) were used to determine significant differences. Comparisons of binary and categorical patient characteristics between subgroups were performed by the use of the Chi-Square test. *P* value < 0.05 was considered statistically significant. All statistical computations were performed with the statistical software environment R, version 3.1.0 (http://www.r-project.org/) or GraphPad Prism Version 6.01.

## SUPPLEMENTARY FIGURES AND TABLES


