# Supplementary material for: Loss of ATRX, associated with DNA methylation pattern of chromosome end, impacted biological behaviors of astrocytic tumors
Source: Oncotarget. 2015 May 4;6(20):18105–15. doi: 10.18632/oncotarget.3906 (PMC4627238; doi:10.18632/oncotarget.3906)
Supplement: Supplementary file 1 [file oncotarget-06-18105-s001.pdf]

SUPPLEMENTARY FIGURES AND TABLES

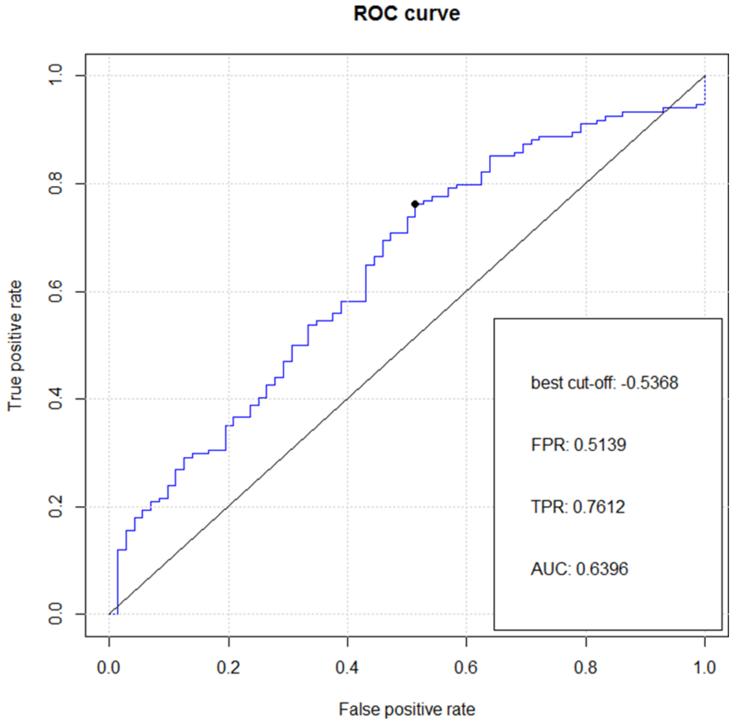

Supplementary Figure 1: The *ATRX* expression threshold was determined by receiver operating characteristics (ROC) analysis with an area under the curve of 0.6396.

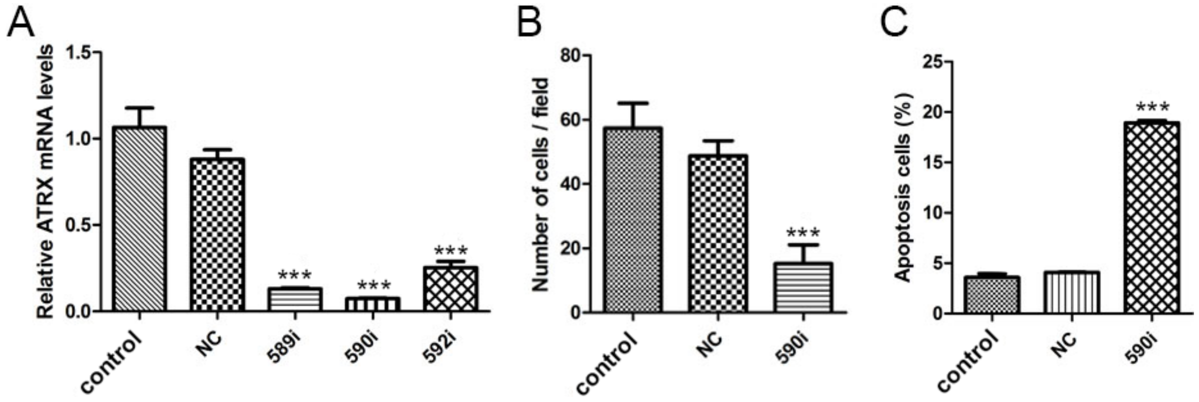

Supplementary Figure 2: The knockdown of ATRX affected migration and apoptosis of LN229 cells. Real-time PCR analysis of the *ATRX* mRNA level in the LN229 cells that were untreated or transfected with 590i or negative control **A.** The number of migrated cells in LN229 cells transfected with 590i was greatly reduced contrasted with control and NC **B.** A statistical analysis of the flow cytometry assays of the annexin V and PI levels in the LN229 cells that were untreated or transfected with 590i or negative control **C.** Data are presented as the means ± S.D. from three independent experiments. *$p < 0.05$, **$p < 0.01$ and ***$p < 0.001$.

Supplementary Table 1: Clinicopathological characteristics of 208 patients with astrocytic tumors

Characteristic	*ATRX-low*	*ATRX-high*	*p-value*
Age at diagnosis			
< 45	53	61	< 0.0001
≥ 45	16	78	
Gender			
Male	38	91	0.1726
Female	31	48	
Preoperative KPS score			
≥ 80	39	73	0.05
< 80	16	61	
NA	14	5	
Histology & Grade			
Astrocytoma (A, II)	41	24	< 0.0001
Anaplastic astrocytoma (AA, III)	4	11	
Primary glioblastoma (pGBM,IV)	19	99	
Secondary glioblastoma (sGBM,IV)	5	5	
TCGA Subtypes			
Proneural	31	19	< 0.0001
Neural	12	30	
Classical	7	11	
Mesenchymal	19	79	
Resection			
Total	28	60	1
Subtotal	36	77	
NA	5	2	
Radiotherapy			
Yes	49	106	0.4488
No	15	24	
NA	5	9	
TMZ Chemotherapy			
Yes	26	73	0.0834
No	34	53	
NA	9	13	
IDH1/2 mutation			
Mutation	51	24	< 0.0001
Wildtype	16	114	
NA	2	1	

Abbreviations: KPS, Karnofsky performance status, NA, not available.

Supplementary Table 2: Factors associated with OS in the Cox regression analysis for patients

Variables	*Univariate Cox Regression*			*Mutivariate Cox Regression*		
	HR	***95%CI***	***p value***	***HR***	***95%CI***	***p value***
ATRX	2.332	1.564–3.479	< 0.0001	1.802	1.144–2.839	0.011
Age	2.193	1.552–3.099	< 0.0001	1.852	1.260–2.724	0.002
Preoperative KPS score	0.958	0.947–0.969	< 0.0001	0.957	0.945–0.969	< 0.0001
Resection	1.381	1.014–1.881	0.041	1.423	1.000–2.027	0.05
Radiotherapy	1.691	1.116–2.561	0.013	3.158	1.999–4.990	< 0.0001
Chemotherapy	0.854	0.602–1.213	0.378			
Gender	0.871	0.613–1.238	0.442			

Abbreviations: KPS, Karnofsky performance status; OS, overall survival.
